# The correlation between external apical root resorption and electric pulp test responses: a prospective clinical trial

**DOI:** 10.1590/2177-6709.26.3.e2119389.oar

**Published:** 2021-06-30

**Authors:** Farnaz YOUNESSIAN, Mohammad BEHNAZ, Mohammadreza BADIEE, Kazem DALAIE, Arezou SARIKHANI, Shiva SHEKARIAN, Shahab KAVOUSINEJAD, Asghar EBADIFAR

**Affiliations:** 1 Nova Southeastern University, College of Dental Medicine, Department of Orthodontics (Fort Lauderdale/FL, USA).; 2 Shahid Beheshti University of Medical Sciences, Orthodontic Department, Dental Research Center, Research Institute of Dental Sciences (Tehran, Iran).; 3 Shahid Beheshti University of Medical Sciences, Orthodontic Department, Dentofacial Deformities Research Center (Tehran, Iran).; 4 Shahid Beheshti University of Medical Sciences, Orthodontic Department (Tehran, Iran).; 5 Private practice (Tehran, Iran).

**Keywords:** Root resorption, External apical root resorption, Electric pulp test, Orthodontic treatment

## Abstract

**Objective:**

The current study investigated the correlation between pulpal sensitivity to the electric pulp tester (EPT) and external apical root resorption (EARR) in four types of maxillary anterior teeth of fixed orthodontic treatment patients.

**Methods:**

In this prospective cohort study, 232 anterior teeth of 58 patients (mean age 18.96 ± 6.13 years) treated with fixed orthodontic treatment were examined. The EPT readings were recorded at twelve time points immediately before archwire insertion. Root resorption of four maxillary incisors were measured by means of parallel periapical radiographs at three time intervals (six months interval from the start) through design-to-purpose software to optimize data collection. A multiple linear regression model and Pearson correlation coefficient were used to assess the association of EPT values and observed EARR (*p*< 0.05).

**Results:**

The highest level of EPT measurement was recorded at initial visit, and then there was a decreasing trend in EPT level during treatment for the next six and twelve months. There was another increasing trend after six months till the finishing time of the treatment. There was a significant correlation between changes in root length and time of recording the root length (*p*< 0.001). There was significant positive correlation between changes in EPT level and amount of observed root resorption (*p*< 0.001).

**Conclusion:**

The relative decrease in electric pulp test level could be a diagnostic sign of root resorption during orthodontic treatment. Further studies with longer follow up are needed to confirm the current results.

## RESUMO

**Objetivo:**

O presente estudo investigou a correlação entre a sensibilidade pulpar ao teste pulpar elétrico (TPE) e a reabsorção radicular apical externa (RRAE) nos quatro dentes anteriores superiores de pacientes em tratamento ortodôntico com aparelho fixo.

**Métodos:**

Nesse estudo de coorte prospectivo, foram avaliados 232 dentes anteriores de 58 pacientes (idade média 18,96 ± 6,13 anos) tratados com aparelho ortodôntico fixo. As leituras do TPE foram registradas em doze tempos de avaliação, imediatamente antes da inserção dos arcos. As reabsorções radiculares dos quatro incisivos superiores foram aferidas por meio da média das radiografias periapicais, em três intervalos de tempo (em intervalos de seis meses desde o início) por um *software* desenvolvido para essa finalidade, com o intuito de otimizar a coleta de dados. Para aferir a associação entre os valores de TPE e as RRAE observadas (*p*< 0,05), foram utilizados o modelo de regressão linear múltiplo e o coeficiente de correlação de Pearson.

**Resultados:**

O nível mais alto das medidas do TPE foi registrado na primeira visita e, daí em diante, houve uma tendência de diminuição no nível do TPE durante o tratamento, nos seis a doze meses seguintes. Houve, também, outra tendência de aumento após os seis meses até o término do tratamento, além de uma correlação significativa entre as mudanças no comprimento radicular e o tempo de registro do comprimento radicular (*p*< 0,001), bem como uma correlação positiva significativa entre as mudanças no nível do TPE e a quantidade de reabsorção radicular observada (*p*< 0,001).

**Conclusão:**

Uma diminuição relativa no nível de sensibilidade ao teste pulpar elétrico pode ser um sinal diagnóstico de reabsorção radicular durante o tratamento ortodôntico. Estudos futuros com acompanhamentos mais longos são necessários para confirmar os presentes resultados.

## INTRODUCTION

External apical root resorption (EARR) occurring in permanent teeth during comprehensive orthodontic therapy is a common iatrogenic phenomenon.^1^ Histologically, orthodontically induced external apical root resorption has been reported to occur with an incidence greater than 90%. However, the radiographic incidence is lower, at approximately 48-66%.^2^ The underlying causes of this unwanted process can be divided into two broad categories, biological and mechanical aspects.^2^ Mechanical factors include orthodontic treatment-related risk factors, such as treatment duration, magnitude of applied force, direction of tooth movement, amount of apical displacement, and method of force application.^3^ The maxillary incisors are the teeth most affected by root resorption, followed by the mandibular incisors and first molars. EARR occurs in different degrees of severity. Severe EARR is defined as a shortening greater than 4 mm or one-third of the root length, and is observed in 1-5% of teeth.^3^

3D imaging (CBCT) has shown to be radiographically valid and reliable in the assessment and diagnosis of EARR. However, considering the potential radiation risks of 3D imaging, the most common diagnostic strategies for root resorption remains 2D conventional images, as panoramic and periapical radiographs, and lateral cephalometries.^4^ Panoramic and lateral cephalometries have been proposed to be more applicable for measurement of EARR, considering their significant advantages of less radiation exposure, visualization of the complete dentition, less time-consuming for the operator, and more patient-friendly, compared to very recent micro-CT three-dimensional methods.^5^ However, they are still considered to be less accurate than periapical films, and overestimate the EARR by 20% the amount of root loss.^6^ ^,^ ^7^ Furthermore, periapical films are prone to magnification errors. According to the recent study by Pereira et al,^8^ this magnification error may be overcome by using the percentage of root/tooth variation, instead of direct measurement of root resorption. Recent advances in the digital image processing and artificial intelligence techniques have made it possible for the computer-assisted superimpositions to be done more accurately, and have increased their clinical applicability. If any root resorption is diagnosed, an inactive phase of 4 to 6 months before the resumption of orthodontic treatment is currently advocated.^5^ Unfortunately, early detection of this condition could not be accomplished before six months from the beginning of resorption.^9^

To date, many studies have evaluated the effect of orthodontic forces on the dentin-pulp complex.^10^ ^,^ ^11^ Different pulp tests have been used in the diagnosis of pulp status.^12^ Electrical pulp test is a simple, noninvasive test that can be used as a sensitivity test for detection of pulp status.^13^ Several studies investigated the correlation between the health of pulpal tissue and the presence of external apical root resorption, with or without orthodontic treatment. Available literature on predisposing higher chance of external apical root resorption in teeth with history of trauma demonstrated the possibility of cause and effect relationship between pulpal tissue and EARR. Therefore, there may be a relationship between electric pulp test (EPT) response of pulp condition and early diagnosis of root resorption. The purpose of the present study was to determine the correlation between orthodontically-induced external apical root resorption and electrical pulp test response of anterior teeth during fixed orthodontic treatment.

## MATERIAL AND METHODS

### SAMPLES AND SAMPLE SIZE CALCULATION

In this prospective non-controlled cohort study, 58 patients (42% male; age range 12-35 years; mean age 18.96 ± 6.13 years) who were referred to the orthodontic department of School of Dentistry, Shahid Beheshti University of Medical Sciences, were selected using random-cluster sampling method. Sample size was determined to be equal to 50 patients (200 anterior teeth sample size) considering α = 0.05, β = 0.20 (power equal to 0.80) and r = 0.2 (low effect size), using sample size calculation software v. 3.0.43. Considering the possibility of dropouts to be 20% during the study, we enrolled 58 patients with 232 anterior teeth. 

#### Inclusion and exclusion criteria

Eligible patients were defined as a minimum of 12 years of age at treatment onset, and treated with multibonded Roth appliances with 0.022 × 0.028-in brackets (3M, Unitek, Monrovia, CA, USA), using the following archwires sequence: 0.016-in round NiTi, 0.016-in round stainless steel, and 0.017 x 0.025-in stainless steel (American Orthodontics, Sheboygan, WI, USA). 

The exclusion criteria were the presence of congenital, systemic or concomitantly diagnosed serious medical conditions, allergy, asthma, familial dysostosis and also history of dental trauma before or during the study, history of lingered pain to thermal stimuli, open apex, any medication, history of previous root resorption, extraction treatment regimen (crowding greater than 7mm in any arch) and also presence of parafunctional habit. Those whose radiographs lacked visibility of maxillary incisors, those with significantly distorted radiographs, crowding of teeth (greater than 7mm in any arch), unclear roots and those with unilaterally and bilaterally lateral missing teeth in the maxilla were also excluded. Those patients who missed to attend their regular monthly follow up or diagnosed with other treatment plans like removable appliances or orthognathic surgery were also excluded from the study. Healthy periodontium (probing depths not exceeding 3 mm, no bone loss as determined by radiographs) and dentition (no carious lesions, no endodontically treated maxillary incisors and closed apex) were necessary to enroll in this study.

All the related demographic data of the patients were recorded. Written informed consent was obtained from the patients and parents for the entire process of the study, including EPT evaluations. The study protocol was based on the ethical principles governing medical research and human subjects in Helsinki Declaration (2013 version, http://www.wma.net/en/30publications/10policies) and also approved by the Research Ethics Committee of Research Institute of Dental Sciences, Shahid Beheshti University of Medical Sciences (ID #3509).

#### Examination of periapical radiographs

The original periapical radiographs of all three groups were obtained with the same digital X-ray unit (KODAK 2100 Intraoral X-Ray, at the same distance and using the same exposure settings: 60 kvp, at 7 mA, 0.2 s). All radiographs were exported and saved in JPEG format using the Digora^®^ software, v. 2.8. The digital radiographs were then visualized and analyzed using Photoshop CS (Adobe Systems Inc., San Jose, CA, USA). A magnification of up to 150% was applied when necessary. All the periapical radiographs were taken in natural head positions, and the examiners used the scanned version of the radiographs.

#### Root resorption assessment

**Figure 1:**
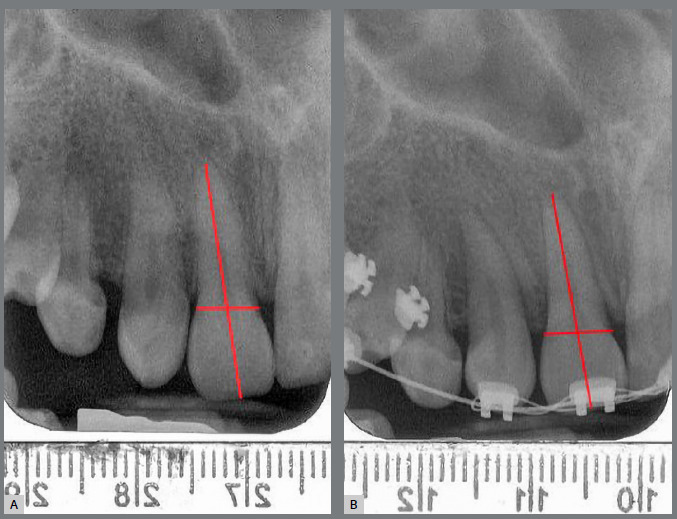
Evaluation of EARR: A) Point selection on an incisor, at the treatment onset; B) Point selection on the same tooth, at the end of fixed orthodontic treatment.

All parallel periapical radiographs were taken with the same device in six months’ time intervals (at the treatment onset and every six months till the first year). To assess the amount of root resorption in the maxillary incisors for each subject, all radiographs were scored by an examiner trained for point registration and blinded to EPT values. In order to measure the distances, a proprietary tool was developed on MATLAB’s image processing toolbox (MATLAB 7.14 2012a, Mathworks Inc, MA, USA). Using this tool, the operator marked three points including root apex, mesial and distal crown edges at the level of cementoenamel junction (CEJ) on the target tooth in both pretreatment and post-treatment X-rays. A perpendicular line was drawn from the apex to the middle of a line passing from mesial to distal points. The incisal midpoint was defined by the intersection of this perpendicular line on incisal edge of each tooth (Fig 1). The root resorption was then calculated automatically by the software, based on the formulation presented by Pereira et al.^8^

To standardize the parallel periapical radiographs, a correction factor was calculated based on the assumption that the crown length had to remain unchanged. Therefore, the ratio C_1_/C_2_ (pretreatment crown length [C_1_]/post-treatment crown length [C_2_]) could determine the inconsistency between crown lengths of the two X-rays, and was used to compensate for the enlargement factor. Apical root resorption was then calculated as follows:

$$\text{CF}={\text{C}}_{1}/{\text{C}}_{2^{\prime }}$$ (1)

$$CF=C_{1}/C_{2},$$ (2)

$$\;Root\;resorption\;=1-\left(CR_{2}/R_{1}\right),$$ (3)

Where CF is the correction factor, C_1_ and R_1_ are the crown length and root length in pretreatment X-rays, while R_2_ and CR_2_ are root length and corrected root length in post-treatment X-rays, respectively. The point marking process was done on an enlarged version of the X-ray, to help reduce the error. Furthermore, the operator repeated the markings five times on each pair of X-rays, recorded the software output after each marking, and used the averaged value as the final root resorption. 

#### Electric Pulp Test (EPT)

Electrical stimulation was provided by the digital electrical pulp tester (Parkell, Farmingdale, NY, USA; 0-80) with toothpaste (Oral B laboratories, Aylesbury, Bucks, England) used as the conduction medium. Examination procedures were performed by the same operator and same EPT unit at each time point. The test of electrical stimuli was applied to the experimental maxillary central and lateral incisors.

To prevent any temperature change and false responses, patients were asked to not eat or drink ten minutes before each visit. After removal of orthodontic archwires, every tooth was isolated with cotton rolls and dried thoroughly before EPT evaluation. The testing site was confined to sound enamel on the midpoint incisal edge of each tooth. This is necessary in order to avoid the orthodontic brackets, and to minimize the risk of false-positive responses elicited by inadvertent stimulation of the periodontal nerve fibers, or stimulation of adjacent teeth. The probe did not touch any orthodontic bands or brackets. Testing of each tooth started upon contact of the smallest electrode tip with voltage 1 on the tooth surface, and terminates when the subjects raised their hands to show feeling of the first sensation (heat or tingling). The EPT has an analog display, providing a score from 01 to 64, correspondent to the stimuli applied. To minimize the procedural errors, a double determination method was used. During testing, current flow was increased slowly from the initial zero current state, by adjusting the variable voltage control. Testing was repeated after a three-minute interval, to reduce subjective fatigue and to minimize the possibility of nerve accommodation. 

The numerical values on the EPT display were recorded at twelve treatment points. The initial EPT levels were recorded exactly before starting the treatment. The EPT scores were also recorded each month immediately before insertion of the new archwire. Teeth that fail to respond to electric testing were recorded as a reading of 0 EPT units. The following clinical and radiologic criteria were used to define pulp necrosis: loss of pulpal sensitivity, gray color changes in the crown, and periapical radiolucency. Loss of pulpal sensitivity and at least one other clinical or radiologic sign were considered necessary before the diagnosis was made. 

#### Statistical analysis

All data were statistically analyzed by SPSS v. 18.0 software (SPSS Inc., USA). To avoid inter-observer error, all the measurements were done by the same operator. In this prospective cohort study, descriptive statistics (including the mean and standard deviations for the EPT response) in experimental group were measured in twelve different time intervals. The observed root resorption were measured by means of periapical radiographs at three time intervals. After all radiographs were assessed, a random subset of ten radiographs was re-examined after fourteen days, to estimate the methodological error by means of percentage of absolute intra-observer agreement (the agreement was 0.81). The EPT values were expressed as mean ± SD. The generalized estimating equation (GEE) with unstructured coefficient and linear equation were used to determine the associations between apical root resorption (EARR) and EPT values status, presented as continuous variables. 

## RESULTS

### DESCRIPTIVE DATA

#### Root length alterations

The radiographs of 58 patients (42% male; age range 12 to 35 years; mean age 18.96 ± 6.13 years) participants were evaluated. Two of the samples were excluded and replaced because of unclear periapical radiographs. Mean and standard deviation of the root length measurement is demonstrated in Table 1. Pairwise comparisons of root length alterations were done within different time points, including initial treatment with sixth month, initial treatment time with twelfth month, and also sixth month with twelfth month measures. The amount of root length alterations in all three comparisons demonstrated statistically significant differences (*p*< 0.001). 

**Table 1:** Mean and standard deviation of root length measurements (mm) at three different time points.

Type of teeth	Time point (months)	Mean	Standard deviation
Central incisor	0	14.58	2.60
	6	13.95	2.56
	12	13.61	2.60
	Total	14.05	2.61
Lateral incisor	0	14.50	2.44
	6	13.88	2.38
	12	13.47	2.37
	Total	13.95	2.43
Total	0	14.54	2.51
	6	13.92	2.47
	12	13.54	2.48
	Total	14.00	2.52

**Figure 2:**
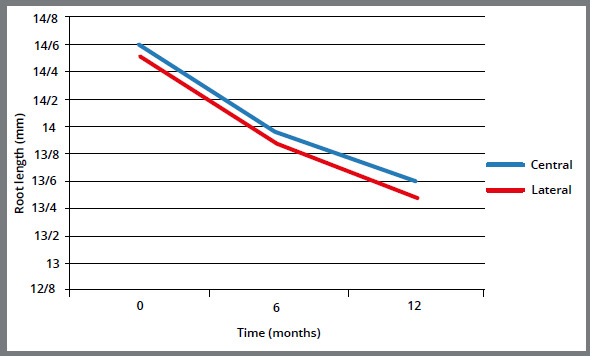
Root length alterations of central and lateral incisors over time.

Root length alterations in both central and lateral incisors also followed the similar pattern. Both teeth demonstrated the maximum root length at baseline and the minimum root length after twelve months. According to Figure 2, the maximum rate of decreasing root length was on the first six months and was similar in both central and lateral incisors (Fig 2). However, the rate of root resorption was higher in laterals in the second six months of the study (time interval 6-12 months). Furthermore, there was a significant correlation between length of the treatment time and root resorption (*p* < 0.001). Based on the measurements, there was about 0.1-mm reduction in root length in each month. Additionally, the type of the tooth (central or lateral) did not have significant effect on the amount of root resorption (*p* = 0.583).

#### EPT levels

**Figure 3:**
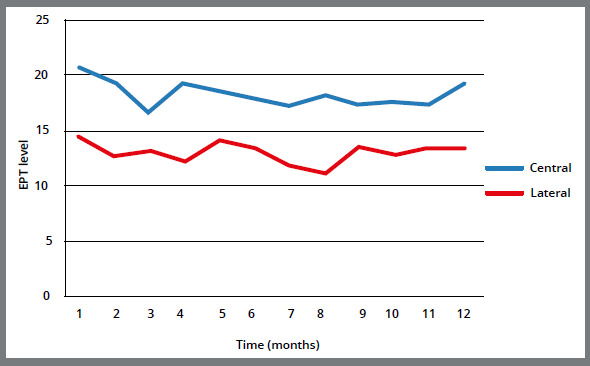
EPT level alterations of central and lateral incisors over time.

EPT changes from T_0_ to T_12_ are demonstrated in Table 2 and Figure 3. The recorded EPT levels were significantly reduced at the first six months, compared to baseline (*p* = 0.007). Comparing the twelfth month with baseline, EPT level was also significantly reduced (*p*< 0.001). However, the reduction in the second six months was not significant comparing to the first six months (Fig 3).

**Table 2:** Mean and standard deviation of EPT measures at twelve different time points.

Type of teeth	Time point (months)	Mean	Standard Deviation
Central	1	14.50	9.97
	2	12.83	9.03
	3	13.35	7.84
	4	12.24	8.16
	5	14.17	11.17
	6	13.54	10.90
	7	11.75	6.77
	8	11.24	6.41
	9	13.51	9.68
	10	12.88	8.83
	11	13.50	8.91
	12	13.45	8.65
	Total	13.08	8.98
Lateral	1	20.68	12.35
	2	19.38	13.23
	3	16.64	9.08
	4	19.42	10.49
	5	18.85	12.56
	6	18.14	11.70
	7	17.12	9.84
	8	18.20	10.92
	9	17.36	10.11
	10	17.65	10.26
	11	17.42	11.24
	12	19.24	10.54
	Total	18.37	11.12
Total	1	17.55	11.61
	6	15.71	11.49
	12	16.22	10.01

The recorded EPT level alterations for central and lateral incisors followed similar pattern. For both type of teeth, the highest levels were recorded in the first visit, and the levels diminished during treatment. Very close to the twelfth month follow up, there was an increasing tendency in the recorded levels. Although the general pattern was the same for both, all the recorded values were significantly smaller in lateral incisors (*p*< 0.001).

#### Correlation of age with root resorption, and age with EPT response

Age did not demonstrate significant correlation with the amount of observed root resorption (*p =* 0.497). Therefore, the selected age range in this study (mean age 18.96 ± 6.13 years) has no statistically significant effect on root length alterations in time. The correlation of EPT values with age demonstrated a significant statistically reverse correlation (*p* > 0.001). Regarding the findings, with each unit increase in age (one year), the EPT level response decreased about 0.86 unit (Table 3).

**Table 3:** Correlation between EPT response and tooth length changes.

Variable	Correlation coefficient *B*	Standard error	*p* **value**
Fixed value of model	14.517	0.5036	0.001
Treatment time	-0.094	0.0084	0.001
EPT	0.016	0.0041	0.001
Type of tooth	-0.028	0.1746	0.872
Age and root resorption	0.022	0.03	0.497
Age and EPT	-0.861	0.1407	0.001

#### Correlation of EPT response, treatment duration and type of tooth with root resorption

The generalized estimating equation (GEE) with unstructured coefficient and linear equation were used to determine the associations between apical root resorption and EPT values. EPT demonstrated statistically significant correlation with the amount of observed root resorption (*p*< 0.001). The correlation coefficient was 0.016 in regression model (standard error of 0.0041), explaining that with each unit reduction in EPT level, 0.02-mm reduction would be expected in root length (Table 3). 

## DISCUSSION

External apical root resorption (EARR) is a common adverse outcome of orthodontic tooth movement. This unwanted phenomenon is seen mostly in teeth that underwent heavy orthodontic forces for long period of time. EARR is also seen in teeth with weak periodontal support.^14^ Various factors including biological and mechanical elements are responsible for the initiation of EARR and its progress during orthodontic tooth movement.^15^ ^,^ ^16^ The most common teeth to undergo EARR are the maxillary incisors and maxillary and mandibular canines, with the average amount of 0.2 to 2.93 mm. The possible controversy regarding the average amount of EARR between studies is mostly due to difference in sample size, individual patient characteristics, type of the tooth considered for the study, type of orthodontic tooth movement, and the measurement method. Different measurement techniques include lateral cephalometry, panoramic view, bisect and parallel periapical radiographs. Parallel periapical radiographs were used in this study at three time points, including before starting treatment, six months, and twelve months after treatment onset. The advantage of using parallel periapical radiographs is the possibility of using fixed and reproducible landmarks, dramatically reducing the subjectivity of the analysis of the level and degree of resorption. The error level of periapical radiographs is four times less than cephalometry radiographs.^17^

Evaluation of the root length in three different time intervals demonstrated that there was a significant reduction in the root length at both time points, compared to baseline (*p*< 0.001). The root length also was significantly reduced between the sixth and twelfth months of study (*p* < 0.001). As orthodontic tooth movement creates inflammatory responses in bone and periodontium, the release of some inflammatory cytokines, including prostaglandins and leukoterins, will occur and increase the possibility of root resorption.^18^ These cytokines increase the vascularity of the regions under orthodontic force. Therefore, pre-osteoclasts and pre-cementoclasts may be created and migrate to the area through the RANK-RANKL pathway. Although osteoclasts and cementoblasts are very similar, cementoclasts are smaller in size and have less number of neuclous .^18^ Previous study of Levander et al^19^ evaluated the risk of root resorption of maxillary incisors during orthodontic tooth movement. Root resorption of 390 upper incisor teeth was evaluated at four time points, including baseline, six months, nine months after treatment onset, and after debonding, using periapical parallel radiographs. The result of the mentioned study demonstrated high risk of root resorption in 6-9 months after initiating orthodontic treatment.^19^ The authors did not report any severe orthodontic root resorption after finishing orthodontic treatment. Another study done by Ravanmehr et al^20^ evaluated the amount of external apical root resorption in time periods of baseline, six, and twelve months. The result of the study was in accordance with the present study, demonstrating significant reduction in root length at six and twelve months following orthodontic treatment. 

The current study evaluated root length alterations based on the type of teeth (central/lateral). The result of this assessment demonstrated similar pattern of root length reduction in both tooth types. Additionally, the tooth type did not show a significant effect on the amount of observed root resorption (*p*= 0.583). This result is in contrast with the result reported by Beck et al,^21^ that found an increased root resorption in lateral incisors. However, in Beck’s study, panoramic radiographs were used instead of periapical radiographs. Krieger et al^22^ also did not report any significant difference between the amount of root resorption and tooth type, using panoramic radiographs. The difference in tooth root morphology was not evaluated in any of these studies.

The maximum rate of the root length changes was reported in the first six months period of fixed orthodontic treatment. However, there was no statistically significant difference between central and lateral teeth. The overall rate of root length alteration was reduced in the second six months period, although lateral incisors demonstrated higher rate of root length alteration in this time period (6-12 months). This study reported the overall rate of 0.1-mm root length reduction per month in these teeth. These reports could be clinically significant in orthodontic patients, and help the clinicians to control or reduce the implemented force to prevent further root resorption in such cases. 

The effect of orthodontic force on dental pulp is evaluated in various studies in the literature.^23^ There is no general consensus regarding the effect of orthodontic forces on dental pulp, and these contradictory results could be due to differences in sample size and type of tested tooth.^13^ ^,^ ^24^ Additionally, evaluation of dental pulp necrosis requires a histopathologic evaluation that is not clinically possible for most cases. Another method is using an electric pulp test (EPT), which is a non-invasive, easy and cost-effective method to evaluate nerve response to orthodontic tooth movement.^24^ This method was used to evaluate the status of pulps in this study. The descriptive results on the changes of EPT values demonstrated a significant reduction in EPT values from baseline to sixth and twelfth months. However, the EPT values did not show any significant change between six to twelve months periods of time. In another study, Hall et al ^25^ evaluated the alteration in EPT values from the baseline, immediately after starting, and at four and eight weeks after treatment. EPT values and temperature testing (cold/hot) were used to evaluate the effect of orthodontic forces.^25^ Based on the reported results, the EPT values were reduced in time, which was in accordance with the result of the current study. In the reported study, the ratio of no response to EPT compared to temperature responses was higher.^25^ Han et al^10^ evaluated the effect of orthodontic forces on EPT responses before, immediately after bonding, and also eight weeks after starting the orthodontic force. They reported an immediate increase in EPT levels response following orthodontic force.^10^ This result is in contrast to the present study, in which reduced EPT level responses were observed. This difference could be due to reduced sample size and shorter periods of follow up. However, the sensitivity of teeth to thermal and EPT was reduced after eight weeks, which is in accordance with the result of previous studies and also the current study.^26^

For Han et al,^10^ the maximum level of reported EPT levels were at the eighth week. However, the maximum level of EPT was immediately before bonding in the present study. This difference could be attributed to different follow up intervals and the type of tested teeth. Modaresi et al^13^ evaluated the effect of orthodontic forces on tooth responses to electric pulp test during 1-month time interval. They reported a significant increase in EPT response threshold immediately after orthodontic loading in maxillary incisors. The EPT responses level decreased after one month of starting orthodontic tooth movement. Two studies also evaluated the effect of orthodontic force on EPT response in nine months, and their finding was the gradual decrease in EPT level in time^10^ ^,^ ^27^ which is in accordance with the result of the current study. However, the follow up time interval was less than the currently tested.

The mechanism underlying these changes includes the alteration in vascularity following orthodontic tooth movement and consequently the presence of transient hypoxia in dental pulp tissue.^28^ This hypoxia could affect *Aδ* and *Aβ* nerve fibers and therefore the pulp response to EPT would change.^13^ Alteration in EPT levels demonstrated similar patterns in both central and lateral teeth. The lowest levels of EPT responses were recorded at the third and eighth months for laterals and centrals incisors, respectively. Regarding previous studies, it seems that the response of different teeth to EPT depends on the type of the teeth. As lateral incisors are subjected to longer and heavier forces during orthodontic treatment, they demonstrated the maximum effect of orthodontic forces. Lateral incisors also are reported to have the maximum amount of root resorption during orthodontic treatment.^29^ Different hypotheses include canine guidance theory, high frequency of crown size and shape malformation, and consequently higher manipulation with orthodontists, high frequency of root shape anomalies like dilacerated and pointed roots, and also smaller root surface area had been mentioned in the literature for this observation.^30^ This study was the first clinical study evaluating the association between EPT responses and root resorption in maxillary incisors. According to the results of the study, for each unit reduction in EPT, a response of 0.02-mm reduction in root length is expected. The clinical application of this study is that without any evidence-based risk factor of root resorption, clinicians could use EPT records as diagnostic tool, to prevent severe root resorption before being evident in panoramic radiographs. 

Further studies with larger sample size and follow up until conclusion of orthodontic treatment are needed to confirm the current results. As EPT only provides information on the status of pulpal nerves, and does not directly determine the vitality (vascularity) of pulp, it is also suggested to repeat the study with vascular measurement techniques, instead of nerve response measurement, which could be a more valid measure to be attributed to root resorption sequel in teeth undergoing orthodontic tooth movement.^28^ Additionally, considering that the EPT evaluation is non-invasive cost effective pulp evaluation, subgroup analysis also is recommended in future studies in case of having large pool of samples based on the presence of various risk factors of root resorption, degree of perceived pain, different morphology of root, different teeth, different treatment regimens (extraction *versus* non extraction). Finally, having access to CBCT radiographs, with significant reduced exposure dosage, would change the ideal measurement tool of EARR in orthodontics.

## CONCLUSION

1. Root resorption was observed in all three-time intervals, and demonstrated a constant increase during twelve-months follow up.
2. The highest level of EPT response was at the first visit and then reduced over time, with slight increase in last months.
3. There was no significant association between type of tooth and observed root resorption, however the association between EPT level change and root resorption was significant.
4. The association between root resorption and EPT levels demonstrated that for each unit reduction in EPT level, a 0.02-mm root resorption was observed.
